# Transcriptome sequencing of lentil based on second-generation technology permits large-scale unigene assembly and SSR marker discovery

**DOI:** 10.1186/1471-2164-12-265

**Published:** 2011-05-25

**Authors:** Sukhjiwan Kaur, Noel OI Cogan, Luke W Pembleton, Maiko Shinozuka, Keith W Savin, Michael Materne, John W Forster

**Affiliations:** 1 Department of Primary Industries, Biosciences Research Division, Victorian AgriBiosciences Centre, 1 Park Drive, La Trobe University Research and Development Park, Bundoora, Victoria 3083, Australia; 2 Department of Primary Industries, Biosciences Research Division, Grains Innovation Park, Horsham, Victoria 3401, Australia; 3 La Trobe University, Bundoora, Victoria 3086, Australia

## Abstract

**Background:**

Lentil (*Lens culinaris *Medik.) is a cool-season grain legume which provides a rich source of protein for human consumption. In terms of genomic resources, lentil is relatively underdeveloped, in comparison to other Fabaceae species, with limited available data. There is hence a significant need to enhance such resources in order to identify novel genes and alleles for molecular breeding to increase crop productivity and quality.

**Results:**

Tissue-specific cDNA samples from six distinct lentil genotypes were sequenced using Roche 454 GS-FLX Titanium technology, generating c. 1.38 × 10^6 ^expressed sequence tags (ESTs). *De novo *assembly generated a total of 15,354 contigs and 68,715 singletons. The complete unigene set was sequence-analysed against genome drafts of the model legume species *Medicago truncatula *and *Arabidopsis thaliana *to identify 12,639, and 7,476 unique matches, respectively. When compared to the genome of *Glycine max*, a total of 20,419 unique hits were observed corresponding to c. 31% of the known gene space. A total of 25,592 lentil unigenes were subsequently annoated from GenBank. Simple sequence repeat (SSR)-containing ESTs were identified from consensus sequences and a total of 2,393 primer pairs were designed. A subset of 192 EST-SSR markers was screened for validation across a panel 12 cultivated lentil genotypes and one wild relative species. A total of 166 primer pairs obtained successful amplification, of which 47.5% detected genetic polymorphism.

**Conclusions:**

A substantial collection of ESTs has been developed from sequence analysis of lentil genotypes using second-generation technology, permitting unigene definition across a broad range of functional categories. As well as providing resources for functional genomics studies, the unigene set has permitted significant enhancement of the number of publicly-available molecular genetic markers as tools for improvement of this species.

## Background

Until recently, strategies for improvement of many global food legume species have been hindered by a lack of genetic and genomic resources. During the early 1990s, barrel medic (*Medicago truncatula *Gaertn.) and *Lotus japonicus *L. were selected as candidate model legume species, due to relatively small genome sizes, inbreeding reproductive habits and short life-cycles [1,2]. Whole genome sequencing projects have been undertaken for these species, providing the opportunity to identify putative orthologous gene sequence resources in other crop legume species, especially those located within the Galegoid clade of the Fabaceae sub-family Papilionoideae [3]. In addition, a draft genome sequence has been completed for the warm-season food legume, soybean (*Glycine max*), which is located in the other major (Phaseoloid) clade http://www.phytozome.net/soybean, providing additional further insights into comparative genomics within the Fabaceae family.

The current dearth of genomic resources for many crop legume species prohibits effective exploitation, through comparative or translational studies, of molecular genetic tools generated from the three genome draft sequences. There is consequently a pressing need for significant efforts to either develop markers capable of cross-species transfer, in order to enrich existing genetic maps, or generate more informative species-specific genetic and genomic tools which can enable the identification of orthologous genes through genome synteny analysis [3].

Lentil (*Lens culinaris *ssp. *culinaris*) is an important grain legume species cultivated throughout Western Asia, the Middle east, North Africa, the Indian subcontinent, North America and Australia, providing a vital source of dietary protein in human diets and straw for animal feed. Lentil is a diploid (2n = 2 × = 14), annual flowering self-pollinating crop with a genome size of c. 4 Gbp [4]. Lentil shares the ability to fix atmospheric nitrogen with other legumes, making it important in the management of soil fertility in cereal based cropping systems. Lentil also provides rotational benefits for management of weeds, diseases and pests, and in many cases offers a profitable, high value crop option for farmers [5]. However, relatively few genomic resources are currently available for lentil, a total of 9,513 EST sequences being present in the public domain as of 3^rd ^February 2011.

Estimation of elapsed times since species divergence from a common ancestor is important for plant comparative genomics. The Galegoid sub-family Vicieae, which contains *M. truncatula*, lentil, field pea (*Pisum sativum *L.) and faba bean (*Vicia faba *L.), diverged from the Loteae sub-family (which contains *Lotus japonicus*) c. 25 million years ago [6]. Despite this extended period of divergence, high levels of macrosynteny are observed between the various Galegoid species [2]. Close genomic relationships have also been observed for more distant comparisons, such as between *Glycine *and *Medicago *[7], for which substantial regions of almost perfect colinearity have been observed. Comparative sequence studies with genome-sequenced legumes is hence of potentially high value for underdeveloped species such as lentil.

Due to recent advances in sequencing technology it has become possible to rapidly generate large datasets with significantly reduced time and labour requirements [8,9]. These methods offer a cost-effective means to access the gene space of a target organism through in-depth sequencing of the transcriptome. Initial transcriptome sequencing studies were largely exploratory, and failed to exploit the potential for next-generation transcriptome sequencing at different scales [10]. However, many recent reports have been published on massively parallel approaches to transcriptome sequencing [11-13], largely using model organisms with available draft genomes to assist assembly [14,15]. Nonetheless, successful studies have also been performed to generate *de novo *transcriptome data assemblies for organisms with no prior genomic resource development [16].

Given that large numbers of plant species are candidates for DNA sequencing, and that technology resources are currently finite, careful decisions are required in respect of sequencing strategy [17], especially for species such as lentil which possess large nuclear genomes. ESTs are single-pass, partial sequences from cDNA clones that provide a rapid and cost-effective method to analyse transcribed portions of the genome, while avoiding the non-coding and repetitive DNA components which contribute the majority of nuclear DNA content in species with large genomes. EST sequencing has proved useful in accelerated gene discovery, including identification of gene family structure, expression analysis, determination of phylogenetic relationships, development of molecular markers such as SSRs and single nucleotide polymorphisms (SNPs) [14,18]. Among the different varieties of molecular genetic markers, SSRs have many advantages including high prevalence and ubiquitous distribution within genomes, co-dominant multi-allelic nature and high reproducibility of assay conditions [19]. Functionally-associated EST-SSRs provide an effective means of molecular marker development that targets nucleotide diversity in genic regions, allowing the possibility of 'perfect' marker development for the molecular breeding of crop plants. In addition, due to location in conserved genic regions, EST-SSR markers frequently display a high degree of operational transferability between related species [20].

This study describes the generation, *de novo *assembly and annotation of a transcriptome-derived EST dataset derived from cDNA obtained from multiple tissues of six genotypes of lentil at various stages of development, using the 454 Life Sciences GS-FLX Titanium second-generation DNA sequencing technology. Clustering and annotation to generate a unigene set has permitted computational identification of SSR loci, and the design and evaluation of a set of EST-SSR marker-directed primer pairs.

## Materials and methods

### Plant material

Lentil seeds were obtained from the Australian Temperate Field Crops Collection held at the Department of Primary Industries, Horsham, Victoria, Australia. Three to four seeds from each of six genotypes (Northfield, ILL2024, ILL7537, ILL6788, Digger, Indianhead), selected based on diversity and important agronomic, abiotic and/or disease trait variation, were sown into commercial potting mix. Germinated plantlets were grown until maturity under glasshouse conditions with natural light on the premises of Department of Primary Industries, Bundoora, Victoria, Australia. Different plant tissues were harvested for RNA isolation from lentil plants at various stages of development, including: leaf (young and mature), stem, flowers, immature pods, mature pods and immature seeds. A total of 10 seeds were also germinated in Petri dishes to harvest seedling root and shoot samples. All of the vegetative plant tissues (leaf and stem) were pooled for RNA isolation and designated LS (leaf/stem) tissue. All of the reproductive organs including flowers, immature pods, mature pods and immature seeds were also pooled for RNA isolation and designated FS (flower/seed) tissue. The seedling-derived root (SR) and shoot (SS) samples were used separately for RNA isolation.

### RNA isolation and cDNA preparation

Total RNA was isolated using the RNeasy mini kit (QIAGEN, Hilden, Germany) following the manufacturer's instructions. Total RNA samples were visually assessed on 1% (w/v) agarose gels for sample integrity before proceeding to cDNA synthesis. cDNA was prepared using SMART cDNA synthesis kit (Clontech Laboratories, California, USA) and mRNA was reverse transcribed by SMARTScribe™ Reverse Transcriptase with the PCR primers SMART IV™ Oligonucleotide and a modified primer with broken chain polyT (5'-AAGCAGTGGTATCAACGCAGAGT CGCAGTCGGTACTTTTTTCTTTTTTV-3') instead of using the standard CDS-III primer [21]. Modification of the cDNA synthesis procedure prevents incorporation of long A/T homopolymer stretches originating from the poly d(A) mRNA tails, which create problems for efficient synthesis during the pyrosequencing reaction. cDNA obtained from LS tissue was normalised using the Evrogen (Sapphire Biosciences Pty Ltd, NSW, Australia) Trimmer cDNA kit to reduce the impact of abundant transcripts which may dominate the generated population of sequence reads. This protocol involves the processes of cDNA denaturation-reassociation, degradation of the double-stranded fraction formed by abundant transcripts using the duplex specific nuclease (DSN) enzyme [22], and PCR amplification of the equalised single-stranded (ss) DNA fraction.

### EST sequence generation, assembly and annotation

cDNA obtained from four different fractions; leaf and stem (LS), flowers, immature pods, mature pods, immature seeds (FS), root germinants (RG) and shoot germinants (SG) were pooled in equimolar ratio before proceeding to GS-library preparation. For ILL2024 (a saline stress tolerant genotype), an additional 10 seeds were germinated in Petri dishes under high salt concentration (80 mM NaCl). Seedlings were used to extract RNA and synthesise cDNA which was then pooled along with other fractions towards the construction of GS FLX library. Approximately 5 mu μg of pooled cDNA was sheared by nebulisation at 206 kPa for 2 min. The GS-FLX Titanium shotgun library construction method was then performed following manufacturer's instructions (Roche Diagnostics). Quantitation of the ssDNA library was performed using real-time PCR and emulsions were prepared using the Lib-L emPCR protocol (Roche Diagnostics, Castle Hill, NSW, Australia). Finally, enriched beads were loaded onto picotitre plates for sequencing.

Primary sequence output has been deposited in the sequence read archive of GenBank (JI846297 - JI861594) prior to assembly. Sequence reads were *de novo *assembled using the Next*Gene *software (Softgenetics, State College, Pennsylvania, USA), adaptor and primer sequences being removed prior to assembly using the 'trimming' function (trim sequences with 100% similarity to the primer/adaptor sequence). *De novo *assembly was performed using the Greedy algorithm and error correction condensation. Assembled contigs and singletons were compared against the *Medicago truncatula *(Mt release 3.0), *Arabidopsis thaliana *(TAIR 9 CDS) and *Glycine max *(Glyma 1.0) transcriptome databases using BLASTN [23] with a threshold E value of 10^-10 ^applied. BLASTN analysis was also performed in the non-redundant database of GenBank using the tBLASTX algorithm to derive putative annotations of the unigene set. Gene ontology (GO) terms were also assigned to the set of unigenes that showed hits against the *Arabidopsis thaliana *database using the 'Gene Ontology at TAIR' tool.

### EST-SSR detection, primer design and marker validation

EST-SSR locus detection and primer pair design was performed using the Batch Primer3 software http://probes.pw.usda.gov/cgi-bin/batchprimer3/batchprimer3.cgi. The parameters were designed for identifying perfect di-, tri-, tetra-, penta-, and hexanucleotide motifs with minimum of repeat numbers of 6, 4, 3, 3, and 3, respectively. Primer design parameters were set as follows: length range = 18 to 23 nucleotides with 21 as optimum; PCR product size range = 100 to 400 bp; optimum annealing temperature = 55°C; and GC content 40-60%, with 50% as optimum.

Lentil genomic DNA was extracted from a total of 13 diverse lentil genotypes (12 from the cultivated species and one non-domesticated related species [*L. nigricans*] for EST-SSR marker validation using the DNeasy^® ^96 Plant Kit (QIAGEN), according to the manufacturer's instructions. Fresh leaf tissue from each genotype was used for each extraction and ground using liquid nitrogen or a Mixer Mill 300 (Retsch^®^, Rheinische Straße, Haan, Germany). DNA was resuspended in 100 μl of water and dilutions performed to a concentration of 10 ng/μl, followed by storage at either -20°C or -80°C. A collection of randomly selected EST-SSR primer pairs were validated experimentally, forward primers being synthesised with addition of an M13 sequence, to enable fluorescent tail addition through the PCR amplification process [24]. PCR conditions included a hot-start at 95° for 10 minutes, followed by 10 cycles of 94° for 30 s, 60-50° for 30 s and 72° for 30 s, followed by 25 cycles of 94° for 30 s, 50° for 30 s and 72° for 30 s and a final elongation step of 72° for 10 min. PCR products were separated using an ABI3730xl (Applied Biosystems, Foster City, California, USA) according to manufacturers' instructions with the addition of the ABI GeneScan LIZ500 size standard and amplification product sizes were determined using the GeneMapper^® ^v3.7 software (Applied Biosystems).

## Results

### Sequencing and *de novo *assembly of EST reads

**Figure 1:**
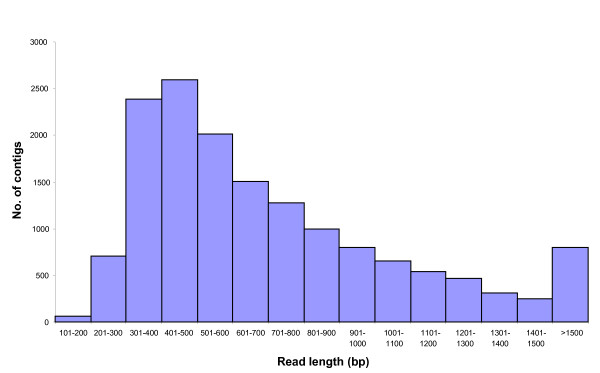
**Frequency histogram depicting the distribution of number of contigs as a function of read length**.

**Figure 2:**
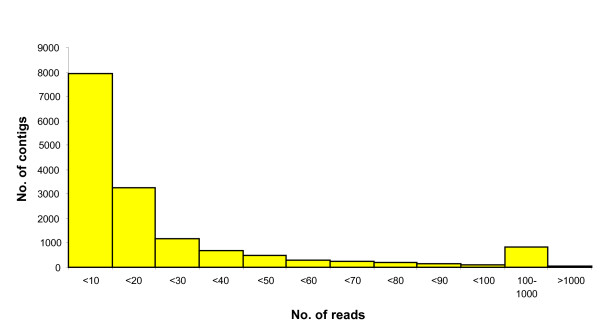
**Frequency histogram depicting the distribution of number of contigs as a function of number of reads**.

A total of 1.38 × 10^6 ^reads corresponding to a cumulative sequence of 448 Mbp were generated from a range of tissues of six genotypes of lentil using the GS-FLX Titanium chemistry. Prior to sequence quality filtering, a median sequence read length of 330 bp was generated. The adaptors, primer sequences and strings of 35 nucleotides from both the 5'- and 3'-termini of each sequence read were removed in order to generate high confidence reads. A total of 847,824 high quality reads were then used to perform *de novo *assembly. After clustering and assembly, a total of 15,359 contigs and 68,715 singletons were obtained, representing a total of 84,074 unigenes (Additional files 1 and 2). The unigene set was then further analysed for quality based on read length, and any remnant sequences less than 100 bp in length were excluded from further analysis, leaving a total of 15,354 contigs and 66,652 singletons. The length of contigs ranged from 114 bp to 6479 bp, with an average of 717 bp. Contig coverage varied from 1.25-fold to 8779-fold, with an average of 13.9-fold. The number of reads per contig varied between 2 and 104,007 with an average of 46 (Table 1). The distributions of read length and number of reads per contig are shown in Figures 1 and 2, respectively. After assembly, 9,614 contigs were identified with read lengths > 500 bp. In addition, only 0.4% of the contigs displayed a read length < 200 bp. This effect might be due to the short length of individual reads, and/or to low coverage of the transcriptome. The majority of contigs (51.5%) were derived from less than 10 reads (Figure 2), followed by 21% composed of up to 11-20 reads per contig. A total of 5.9% of the contigs were composed of more than 100 reads in each contig.

**Table 1:** Summary information for contig assembly of lentil ESTs

Number of reads per contig	Number of contigs	Percentage of total contigs per read number class
2	271	1.8
3	731	4.8
4	1641	10.7
5	1519	9.9
6	1194	7.8
7	1000	6.5
8	870	5.7
9	685	4.5
10	588	3.8
11-15	1798	11.7
16-20	1016	6.6
21-25	632	4.1
26-30	452	2.9
31-35	347	2.3
36-40	312	2.0
41-45	263	1.7
46-50	202	1.3
> 50	1833	11.9

**Figure 3:**
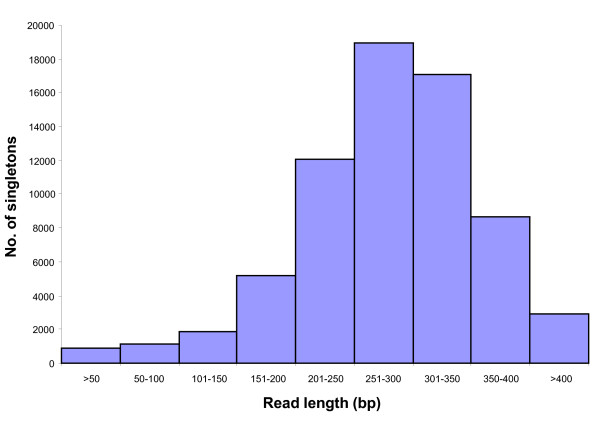
**Frequency histogram depicting the distribution of number of singletons as a function of read length**.

The length of singletons varied from 101-560 bp, with an average of 286 bp. The majority of singletons (72%) were between 200-350 bp in length, and 4.3% were longer than 400 bp (Figure 3).

### Structural and functional annotation of ESTs

The derived unigene set was analysed using BLASTN against the *M. truncatula *genome draft, and a total of 12,639 unique matches were obtained (6,978 contigs and 5,661 singletons) (Additional file 3). All consensus sequences were also compared against the nr database of GenBank. A total of 12,544 contigs and 33,013 singletons (45,557 unigenes) obtained matches at E < 10^-10^. Any query sequences that revealed a most significant match against a non-plant species were removed from the list, leaving a total of 44,756 entries (Additional file 4, sheet 4.1). As more than one contig or singleton can originate from a single gene due to either non-overlapping sequence reads or high levels of sequence error in a single read, it is feasible to generate a collapsed sequence collection for identification of multiple unigenes that originate from the same gene. A cohort of gene loci were identified that corresponded to a set of 25,592 unique matches (10,506 contigs, 41%; 15,086 singletons, 59%) (Additional file 4, sheet 4.2). Base substitution rate was determined through comparison of consensus sequences of orthologues from *Lens culinaris*, *Medicago truncatula *and *Arabidopsis thaliana *for four randomly selected genes (each being > 0.5 kb in length) (data not shown). Average nucleotide substitution rate in the lentil and *M. truncatula *comparison was found to be 9 per 100 bp (stdev = 1.1), but the equivalent value when comparing lentil to *A. thaliana *was 23/100 bp (stdev = 2.6). Due to the relatively high degree of sequence conservation, data from the present study is also applicable to model species such as *M. truncatula *and *A. thaliana*, providing an opportunity to study comparative genomics and evolutionary relationships between dicotyledonous plant species.

**Figure 4:**
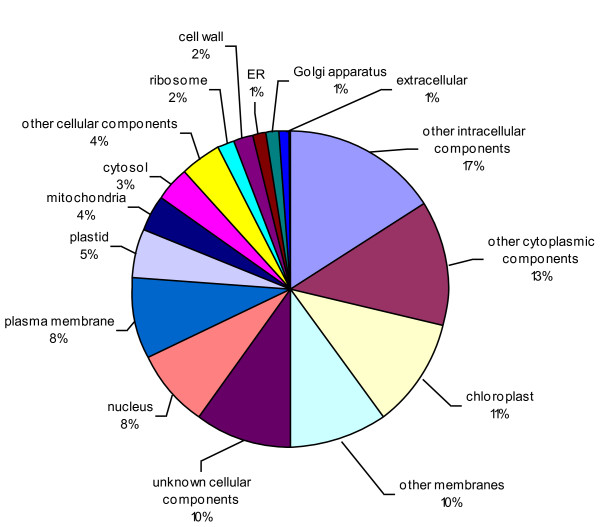
**Pie-chart representation of GO annotation results from lentil consensus sequences for Cellular Component, with a total number of gene counts of 11,446**. Since one gene product can be assigned to more than one GO terms, the total percentage in each category could exceed 100%.

**Figure 5:**
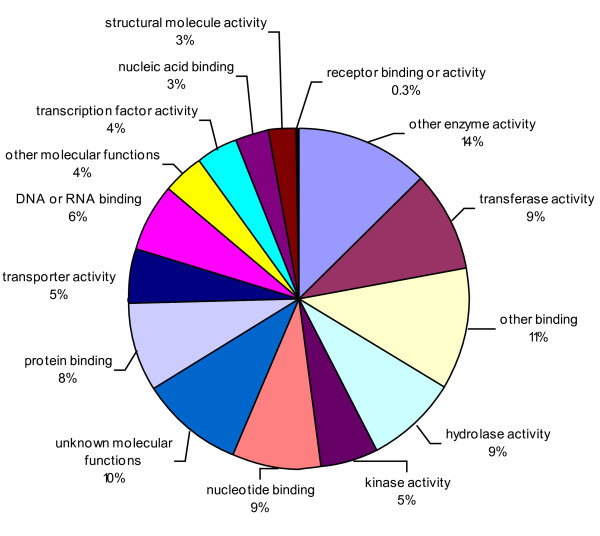
**Pie-chart representation of GO annotation results from lentil consensus sequences for Molecular Function, with a total number of gene counts of 119,316**. Details are as for Figure 4.

**Figure 6:**
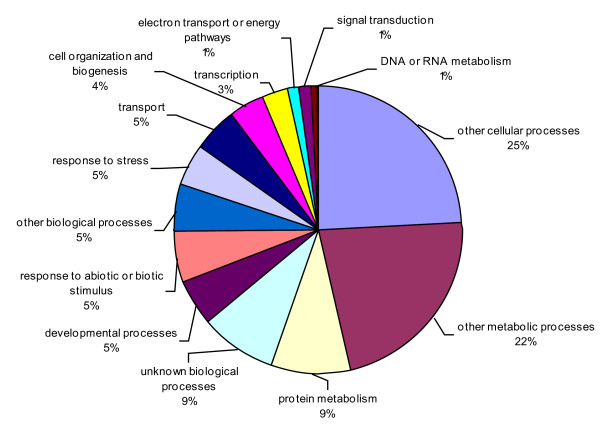
**Pie-chart representation of GO annotation results from lentil consensus sequences for Biological Process, with a total number of gene counts of 13,270**. Details are as for Figure 4.

The consensus sequences were also compared against *Arabidopsis thaliana *database and 7,476 unique matches were identified, including 3,941 contigs and 3,535 singletons (Additional file 5). All unique matches were annotated and gene ontology (GO) terms were further assigned corresponding to a total of 34,034 gene counts and 44,734 annotation counts (Figures 4-6). The intracellular component category of the cellular component classification class contributed the largest proportion of all annotations (17%), followed by the cytoplasmic component (13%), chloroplast component (11%), membrane component (10%), other cellular component (10%), nuclear component (8%) and plasma membrane component (8%) categories. Other components such as plastid, cytosol, mitochondria, ER, golgi apparatus, cell wall, ribosome and extracellular components were represented at proportions less than 5% of total (Figure 4). Among the molecular function classification class, the enzyme activity, transferase activity, binding activity, hydrolase activity, molecular function, nucleotide binding and protein binding categories included the majority of detected matches (Figure 5). In the biological processes classification class, cellular (25%) and metabolic processes (22%) constituted the major categories, followed by protein metabolism (9%), unknown biological processes (9%), developmental processes (5%), stress response (5%), transport (5%) and cell organization and biogenesis (4%) (Figure 6).

Finally the lentil unigene set was also compared against *Glycine max *EST sequences database [http://www.phytozome.net/soybean, 25] that identified 20,419 unique matches (Additional file 6).

### Frequency and distribution of EST-SSRs in lentil transcriptome

EST-SSR discovery was performed based on analysis from assembled contig templates, and a total of 2,929 distinct loci were identified, a frequency of 16% (2,415 SSR containing contigs/15,354 total contigs). A total of 2,393 SSR primer pairs were designed from these loci, 412 template contigs containing at least two SSR loci eligible for primer pair design (Additional file 7). Incidences of different repeat types were determined (Table 2), the most abundant being trinucleotide arrays (1,424: 60.6%). Frequencies for each array type according to repeat unit number were also evaluated (Table 2), the most common class being n = 5 (1,037 loci: 44.1%), while only 1.1% of loci contained more than 9 repeat units.

**Table 2:** Summary information on frequencies of different SSR repeat motif types related to variation of repeat unit numbers in lentil EST-SSR loci

SSR motif length					Repeat unit number						
	3	4	5	6	7	8	9	10	> 10	Total	%
Di	-	-	-	53	25	8	5	1	4	96	4.1
Tri	-	955	296	90	48	18	10	4	3	1424	60.6
Tetra	455	37	7	2	-	-	-	-	-	501	21.3
Penta	141	18	5	1	-	-	-	-	-	165	7.0
Hexa	135	27	3	-	-	-	-	-	-	165	7.0
Total	731	1037	311	146	73	26	15	5	7	2351	
%	31.1	44.1	13.2	6.2	3.1	1.1	0.6	0.2	0.3		

### Validation of a subset of EST-SSRs

A subset of 192 EST-SSR primer pairs were selected for validation of marker assay performance. A total of 166 primer pairs successfully obtained amplification products from one or more template genotype, of which 51 (30.7%) revealed polymorphism between 12 *L. culinaris *genotypes. Inclusion of the non-domesticated species *L. nigricans *permitted polymorphism detection by 28 additional primer pairs (an increase to 47.5% of total) (Additional file 8).

## Discussion

### Assembly and annotation

A limited amount of genomic data pertaining to the cool-season food legume lentil (as well as related species such as chickpea, field pea and faba bean) was publicly available prior to commencement of this study. Relatively few activities have previously been performed to address this deficit. The 454 Life Sciences GS-FLX Titanium second-generation sequencing technology provides a rapid, efficient and cost-effective method for genomic resource enrichment through generation of large numbers of ESTs with individual read lengths of up to 500 bp. The technology has previously been used to perform *de novo *bacterial genome sequencing, whole genome shotgun sequencing, metagenomic studies, transcriptome characterisation and small RNA sequence determination [26].

In order to produce a maximally informative lentil transcriptome sequence resource, cDNA from leaf and stem tissue was normalised prior to sequence analysis. This process reduces oversampling of abundant transcripts such those derived from the chloroplast or nuclear genes involved in photosynthetic processes (e.g. *rbcL*, *rbcS*, *cab*), and more efficient detection of transcripts expressed at low levels in specific tissues. A preliminary study (data not shown) suggested that normalisation of lentil cDNA could enhance rare transcript detection by c. 10%. A similar approach was effective for detection of lowly-expressed genes from cDNA sequencing in both *M. truncatula *and *Artemisia annua *[14,27]. Alternatively, laser-capture microdissection followed by second-generation sequencing may be used for rare transcript identification, but the process is technically challenging [28,29].

The quality of sequence generation, which may be negatively impacted by poly d(A) content, was also improved by use of a modified primer with an interrupted poly d(T) tail, leading to an increase of 5-6% in output of the total number of sequenced fragments (data not shown). Higher levels of enhanced sequencing output may be attainable for full length cDNA transcripts, which were not targeted in the current study. Similar approaches have been taken in other reports to maximise sequencing output [30,31].

Some recent studies have indicated that short reads from 454 GS FLX pyrosequencing may be effectively assembled and used to characterise the gene space in various organisms [16,21,32,33]. The GS FLX Titanium technology upgrade has permitted recovery of read lengths up to 400-500 bp, and hence assembly of larger contigs. The average contig length produced in the present study was 770 bp, which is significantly longer than those derived from previous studies (e.g. 197 bp; [34], 334 bp; [27], 440 bp; [31], 500 bp; [16]). A majority (56.4%) of reads were assembled into contigs, comparable to efficiencies from similar studies (62%, [27]; 48%, [16]). A large number of singletons (68,715) were also obtained, consistent with other studies based on 454 GS technology [16,27]. Singleton generation may be due to a variety of causes such as sequencing error, template contamination and effect of assembly algorithm [35]. It was also noted that most of the singletons were obtained from source genotype ILL2024 under salt stress treatment, which may represent a class of genes that were not expressed in the other genotypes. A total of 48% of singletons matched GenBank accessions, leaving 52% singletons unmatched, this is in contrast to the proportions which failed to match known databases in parallel studies (13%; [16], 32%; [20]). However the 52% of singletons that failed to match any known sequence in GenBank is only 4% (35,702) of the total reads (847,824) that were committed to assembly, which is relatively common in transcriptome sequence analysis. These effects may be due to several possible factors, including the propensity of short read lengths to hinder assembly, and incomplete incidence of genes corresponding to low abundance transcripts in current sequence databases [20]. Among the 33,013 singletons that revealed significant matches against a known database, 699 were annotated against a bacterial, viral, animal or human genome. In addition, many singletons showed matches to the same gene, resulting in 15,086 singletons with unique annotations, which contributed a majority of the c. 25,000 unigenes. This value is comparable to the total number of genes identified in genome drafts of other diploid plant species such as rice, sorghum, *A. thaliana *and *Brachpodium distachyon *[36,37].

Given that next-generation sequencing projects will soon become common in non-model organisms, increases in the amount and quality of derived data should progressively result in the improvement of *de novo *assembly algorithms. In addition, enrichment of genomic resources will further assist in identification of rare transcripts from collections of sequences that include those of low quality. However, a large proportion (81%) of the assembled contigs exhibited significant matches to present versions of public domain sequence databases. This confidence in annotation has presumably been assisted by the improvements of contig length (62% longer than 500 bp) obtained from the use of GS-FLX Titanium chemistry. Estimation of the number of genes and level of transcript coverage represented in an EST collection is an important objective for transcriptome sequencing projects, but is difficult or impossible without a completely annotated reference genome sequence. Assuming a similar number of genes (approx 25,000) as in other diploid plant genomes, the sequences annotated in this study are likely to represent c. 50% of the lentil gene complement. These estimates were supported by comparative analysis with *M. truncatula*, for which 12,639 unique matches represent c. 50% of the known gene space. For more distantly related, *A. thaliana*, the corresponding value was c. 30% of the gene space. In comparison to *G. max*, 20,419 unique hits represent c. 31% of the known gene space (compared with a total of predicted 66,153 protein-coding loci),

### Marker identification and characterization

SSR-based marker systems have increasingly become popular for population genetic analyses and genetic mapping studies [38]. Traditional methods for development of genomic DNA-derived SSRs are expensive, laborious and time-consuming. However, ESTs generated by large-scale transcriptome sequencing programs are a potentially rich source for SSR discovery. EST-SSRs exhibit potential advantages when compared to SSRs located in non-transcribed regions due to generally more consistent efficiency of amplification, and enhanced cross-species transferability [39,40]. Substantial numbers of SSR loci were identified in the present lentil EST collection, supporting high quality PCR primer design in most instances. As EST-SSR loci are genic and have been derived from transcriptome database, the majority of the EST-SSR loci should occur in the protein-coding sequences of annotated contigs, representing genes of known or predicted identity and function.

The frequency of EST-SSR loci detected in cultivated lentil is similar to estimates from other broad-leaved plant species (2% to 17%) [41]. These prevalence values are influenced by the type of software (such as SPUTNIK, SSRIT, Batchprimer3 and FastPCR) and default parameters used for detection, which would be expected to produce minor influences on the efficiency of detection [42-46]. In the present study, trinucleotide units are the most abundant form of SSR repeat structure, consistent with results from other plant species [47,48].

The results of EST-SSR validation using cultivated and non-domesticated *Lens *genotypes suggest that polymorphism frequency is highly enhanced by inclusion of germplasm outside the pooled of contemporary germplasm, as observed for other crop plant species [49-51]. The ability to amplify across specific boundaries is a common property of EST-SSRs, as previously described, and has been demonstrated for other legume taxa through efficient detection of polymorphisms in other *Medicago *species such as alfalfa by *M. truncatula*-derived EST-SSRs [46]. *L. culinaris*-derived SSRs may hence be implemented for study of the genus *Lens *in the broader sense, to fully access exotic gene pools.

## Conclusions

Generation of a substantial EST-derived dataset from cultivated lentil is described in this study, comprising 84,074 unigenes, of which c. 25,000 have been sequence annotated. A set of EST-SSR primer pairs has been designed using unigene templates and demonstrated to be effective for polymorphism detection within cultivated germplasm and across the genus *Lens*.

## Authors' contributions

SK performed the 454 GS FLX sequencing, analysed the EST dataset, designed EST-SSR marker assays, analysed SSR marker validation data and drafted the manuscript. LP assisted in the preparation of cDNA libraries for sequencing analysis. MS screened the EST-SSR marker subset on lentil germplasm. KS assisted the sequence contig annotation process. NC contributed to data interpretation and assisted in drafting the manuscript. JF and MM co-conceptualised and coordinated the project, and assisted in drafting the manuscript. All authors read and approved the final manuscript.

## Supplementary Material

Additional file 1 **Consensus sequences of assembled contigs**. The data represents the consensus sequences of 15,354 assembled contigs generated as a result of *de novo *assembly of lentil ESTs. This number varies from the sequences available via GenBank due to contig length filtering imposed by TSAdb. Click here for file

Additional file 2 **Sequence information of singletons**. The data represents the sequence information on all the singletons generated from *de novo *assembly of lentil ESTs. Click here for file

Additional file 3 **Bioinformatic annotation (BLASTN) of lentil unigene set against *Medicago truncatula *genome**. This file contains the BLAST results obtained as a result of comparison of lentil unigene set against *M. truncatula *genome at an E value < 10^-10^. Click here for file

Additional file 4 **Bioinformatic annotation (BLASTX) of lentil unigene set against nr databse of Genbank**. This file contains the BLAST results obtained as a result of comparison of lentil unigene set against *nr *databse of GenBank at an E value < 10^-10^. Click here for file

Additional file 5 **Bioinformatic annotation (BLASTN) of lentil unigene set against *Arabidopsis thaliana *genome**. This file contains the BLAST results obtained as a result of comparison of lentil unigene set against *A. thaliana *genome at an E value < 10^-10^. Click here for file

Additional file 6 **Bioinformatic annotation (BLASTN) of lentil unigene set against *Glycine max *genome**. This file contains the BLAST results obtained as a result of comparison of lentil unigene set against *G. max *genome at an E value < 10^-10^. Click here for file

Additional file 7 **Sequence information of all of the SSR primer pairs identified and designed using BatchPrimer3**. This file contains all of the information (sequence information, orientation, sequence length, expected product length, expected position on Mt genome, Tm, GC content and SSR motif length) on SSR primer pairs designed using BatchPrimer 3. Click here for file

Additional file 8 **Characterization of a sub set of EST-SSRs on wild and cultivated genotypes of lentil**. The table represents the data on number and size of alleles amplified from screening of 192 EST-SSR primer pairs on 13 genotypes of lentil. Click here for file
